# Effect of autologous platelet-rich plasma on optical quality and visual outcomes following implantation of a trifocal diffractive intraocular lens

**DOI:** 10.1371/journal.pone.0340880

**Published:** 2026-01-27

**Authors:** Kun-Hoo Na, Bo Young Lee, Hye Jin Cho, Joon Young Hyon

**Affiliations:** 1 B&VIIT Eye Center, Seoul, South Korea; 2 Department of Ophthalmology, Seoul National University College of Medicine, Seoul, South Korea; 3 VISUWORKS, Seoul, South Korea; 4 Seoul National University Bundang Hospital, Seongnam, South Korea; BaekSeok University, KOREA, REPUBLIC OF

## Abstract

**Purpose:**

To evaluate the effect of autologous platelet-rich plasma (PRP) eye drops on optical quality and visual acuity in eyes implanted with a trifocal diffractive intraocular lens (IOL).

**Methods:**

In this retrospective cohort study of 128 eyes from 128 patients undergoing femtosecond laser-assisted cataract surgery with implantation of the CNWT Clareon PanOptix (Alcon Inc.) IOL, patients were assigned to one of two groups: the PRP group (n = 62), which received postoperative PRP eye drops, and the conventional treatment group (n = 66). At 3 weeks postoperatively, optical parameters—including the objective scatter index (OSI), modulation transfer function (MTF) cutoff, and Strehl ratio (SR)—were measured with a double-pass system. Uncorrected distance visual acuity (UDVA) and corrected distance visual acuity (CDVA) at 4 m, as well as uncorrected near visual acuity (UNVA) at 40 cm, were recorded in logMAR units.

**Results:**

The PRP group demonstrated superior optical quality across all measured parameters compared to the control group, with lower OSI (1.60 vs. 3.14), higher MTF cutoff (31.93 vs. 19.90 cycles/degree), and higher SR (0.15 vs. 0.12) (all *P* < 0.001). Visual acuity was also significantly better in the PRP group, with lower mean logMAR UDVA (0.05 vs. 0.10; *P* < 0.001), CDVA (0.01 vs. 0.05; *P* < 0.001), and UNVA (0.13 vs. 0.18; *P* = 0.007). Multivariate analyses confirmed that the use of PRP eye drops was independently associated with improvements in all three optical parameters: OSI (B = −1.488), MTF cutoff (B = 12.038), and SR (B = 0.039) (all *P* < 0.001).

**Conclusions:**

The use of autologous PRP eye drops improves optical quality and visual acuity at all distances following trifocal diffractive IOL implantation. PRP eye drops may serve as a beneficial adjunct for postoperative management in patients undergoing this procedure.

## Introduction

As the demand for presbyopia correction and reduced spectacle dependence following cataract surgery grows, multifocal intraocular lenses (IOLs) are increasingly utilized [1]. Among these, trifocal diffractive IOLs have gained popularity due to their ability to provide satisfactory visual acuity across multiple distances [2–4]. However, concerns persist regarding the optical quality of multifocal IOLs compared to monofocal IOLs [5–7]. Given that multifocal IOL implantation is typically performed in otherwise healthy individuals without ocular or systemic comorbidities and that these IOLs incur higher costs, achieving high-quality postoperative optical outcomes holds substantial clinical relevance, particularly in meeting elevated patient expectations [1,8]. Even without overt ocular surface disease, subclinical tear film instability or subtle optical degradation may compromise visual satisfaction [8]. This underscores the relevance of interventions aimed at optimizing optical quality even in clinically healthy eyes**.**

In recent years, there has been growing interest in blood derived products, such as autologous serum (AS) or platelet-rich plasma (PRP), for treating ocular surface diseases [9]. Autologous PRP has attracted particular attention due to its high concentration of biological factors in platelets that promote ocular surface healing [10–12]. It has demonstrated comparable or superior efficacy to AS in managing dry eye disease (DED) and primary Sjögren’s syndrome, with the added benefit of a shorter preparation time [13–15]. Furthermore, PRP has shown therapeutic potential in ocular surface disorders unresponsive to conventional therapies, such as dormant corneal ulcers and persistent corneal epithelial defects [11,12,16,17].

Ocular surface irregularities, especially those associated with DED, have been shown to negatively impact the optical performance of multifocal IOLs. A stable tear film is thus essential for achieving optimal visual outcomes in eyes implanted with multifocal IOLs [8]. Previous studies have identified DED as a significant contributor to patient dissatisfaction following implantation of presbyopia-correcting IOLs [18,19]. Based on this evidence, it is reasonable to hypothesize that PRP eye drops, by promoting ocular surface stabilization and tear film homeostasis, could enhance optical and visual performance in eyes implanted with trifocal diffractive IOLs. To the best of our knowledge, no prior studies have investigated this hypothesis.

In this study, we aimed to evaluate the effects of autologous PRP eye drops on optical quality and visual outcomes following trifocal diffractive IOL implantation by comparing the conventional treatment (CT) group with a PRP group.

## Methods

### Study population

In this retrospective cohort study, we reviewed consecutive patients who underwent femtosecond laser-assisted cataract surgery (FLACS) and implantation of the CNWT (Clareon PanOptix; Alcon Inc., Fort Worth, TX, USA) IOL between August 2022 and August 2023 at B&VIIT Eye Center, South Korea. This study adhered to the tenets of the Declaration of Helsinki and was approved by the Public Institutional Review Board (IRB) designated by the Ministry of Health and Welfare (Protocol number: P01-202405-01-018). The IRB waived the requirement for written informed consent due to the retrospective nature of the study and the use of anonymized data. The data were accessed on May 27, 2024, and the authors did not have access to any identifiable participant information during or after data collection. All data were fully anonymized prior to access.

### Group classification

Patients were divided into two groups: the CT group received the standard postoperative regimen of 0.5% moxifloxacin, 1% prednisolone, 0.45% ketorolac eye drops, and 0.18% preservative-free hyaluronic acid (HA) artificial tears; the PRP group received PRP eye drops in addition to this regimen. Exclusion criteria included corneal astigmatism greater than 1.0 diopter (D), any ocular comorbidities other than cataracts (such as corneal pathology, lid abnormalities, retinal or optic nerve disease), and history of ocular surgery. Patients with diabetes, autoimmune diseases (e.g., Sjögren syndrome, rheumatoid arthritis), psychiatric disorders, thyroid disease, allergic diseases, hematologic disorders, heart disease, acute or chronic infections, history of cancer treatment, or who were undergoing antiplatelet or anticoagulant therapy were also excluded. Additionally, patients who did not attend regular follow-up visits at 1 and 3 weeks postoperatively were excluded.

### Preoperative examinations

All patients underwent comprehensive ophthalmologic evaluations before surgery. Uncorrected distance visual acuity (UDVA), corrected distance visual acuity (CDVA) at 4 m, and uncorrected near visual acuity (UNVA) at 40 cm were measured using Early Treatment Diabetic Retinopathy Study charts and reported in logarithm of the minimum angle of resolution (logMAR) units. Additional examinations included slit lamp biomicroscopy, specular microscopy, corneal topography and tomography, dilated fundoscopy, and optical coherence tomography. Ocular biometry was performed using the IOLMaster 700 (Carl Zeiss Meditec AG, Jena, Germany), and IOL power targeting emmetropia was determined based on the Barrett Universal II formula.

### Surgical techniques and postoperative management

All cataract surgeries were performed under topical anesthesia using the FLACS with the LenSx system (Alcon Inc., Fort Worth, TX, USA) to create a 4.9-mm capsulorhexis and quadrant-pattern lens fragmentation. Phacoemulsification, irrigation, aspiration, and polishing were then carried out with the CENTURION Vision System (Alcon Inc., Fort Worth, TX, USA). The IOL was implanted into the capsular bag, and all incisions were sealed via sutureless stromal hydration. All surgeries were uneventful, and no intraoperative complications—such as posterior capsular rupture, zonular dehiscence, or significant intraoperative miosis—occurred in any case. Postoperatively, patients instilled 0.5% moxifloxacin and 1% prednisolone eye drops four times daily, 0.45% ketorolac eye drops twice daily, and 0.18% HA artificial tears six times daily. Follow-up visits were scheduled at 1 and 3 weeks after surgery.

### Preparation of autologous platelet-rich plasma eye drops

At the 1-week postoperative visit, PRP eye drops were prepared for the PRP group using the 3E-PRP kit (Pervice Co., Ltd., South Korea) according to the manufacturer’s instructions [20]. The manufacturer reports that the final platelet concentration achieved with this method is approximately 9.3–12 times higher than the baseline whole-blood level [20]. We collected 8.5 mL of venous blood into 10-mL sterile tubes containing 1.5 mL of anticoagulant citrate dextrose solution A. After gently mixing, samples were centrifuged at 3,000 rpm for 5 minutes to yield three layers: (1) an upper plasma layer, (2) a middle platelet-rich buffy coat, and (3) a lower red blood cell layer. Using the provided connector, we aspirated the supernatant plasma and buffy coat into separate syringes, combined them to form PRP, and divided the final product into 2 bottles, each containing approximately 2 mL. Patients were instructed to instill PRP drops four times daily for 2 weeks, starting 1 week postoperatively; opened bottles were stored at 4 °C and unopened ones at −20 °C.

### Measurement of optical quality parameters and other postoperative evaluations

Optical quality was assessed at the 3-week postoperative visit using the double-pass optical quality analysis system (OQAS II; Visiometrics, Spain), which evaluates the combined effect of ocular aberrations and intraocular scattering [21–23].This system captures images from a 780 nm point-source object reflected on the retina and directly computes the modulation transfer function (MTF) from the acquired double-pass retinal image using Fourier transformation. It also presents MTF-related parameters, such as the MTF cutoff and Strehl ratio (SR). The MTF cutoff is the highest spatial frequency detectable by the eye, with higher values indicating superior optical quality. SR is defined as the ratio between the area under the MTF curve of the measured optical system and that of an aberration-free system, with higher SR values corresponding to better optical performance [22,24]. Additionally, the system provides the objective scatter index (OSI), which quantifies intraocular light scattering. The OSI is defined as the ratio of light intensity at a peripheral location to the central peak in the double-pass image, with higher OSI values indicating greater intraocular scatter and visual disturbance [22,23,25]. All measurements were performed under mesopic conditions with a 4.0-mm artificial pupil by experienced technicians.

Noninvasive keratograph break-up time (NIKBUT) was measured at 1-week and 3-week postoperative visits using the Keratograph 5M (Oculus, Wetzlar, Germany) [26,27]. Both the time of initial tear break-up (NIKBUT-first) and the average time of all tear break-ups (NIKBUT-avg) were recorded.

UDVA and CDVA at 4 m, and UNVA at 40 cm were measured at the 3-week postoperative assessment. Additionally, total corneal higher-order aberrations (HOAs) at a 6-mm diameter, spherical aberration (SA) at a 4-mm diameter, and the degree of IOL decentration and tilt were evaluated using anterior segment swept-source optical coherence tomography (CASIA2; Tomey, Japan).

### Statistical analysis

Statistical analyses were performed using SPSS for Windows (version 28.0; SPSS Inc., Chicago, Illinois, USA) and R software (version 4.4.0; R Foundation for Statistical Computing, Vienna, Austria). Continuous variables were compared using independent *t*-tests, and categorical variables were analyzed using the chi-square test. Jitter plots were used to visually represent the distribution of optical quality parameters. Univariate and multivariate linear regression analyses were conducted to identify factors associated with OSI, MTF cutoff, and SR. Variables with a *P* value of less than 0.05 in the univariate analysis were included in the multivariate model to assess their independent effects. Multicollinearity was evaluated using the variance inflation factor (VIF), with VIF greater than 10 considered indicative of significant collinearity [28]. A *P* value of less than 0.05 was considered statistically significant.

## Results

### Baseline characteristics

Among the initially enrolled 148 patients, 20 were excluded for the following reasons: previous laser vision correction surgery (n = 4), diabetes (n = 6), rheumatoid arthritis (n = 1), Sjögren syndrome (n = 1), hypothyroidism (n = 1), current use of antiplatelet agents (n = 4), and hepatitis B virus carrier status (n = 3). Consequently, 128 eyes from 128 patients were included in the final analysis, with 62 patients assigned to the PRP group and 66 patients allocated to the CT group.

The mean age in both groups was in the late 50s, and approximately 60% of patients were female. No significant differences were observed between the two groups regarding preoperative visual acuity, spherical equivalent (SphEq), axial length, white-to-white corneal diameter, corneal power, central corneal thickness (CCT), and IOL power (Table 1).

**Table 1:** Demographic and baseline ophthalmologic characteristics.

	PRP group (n = 62)	CT group (n = 66)	*P* value
Age (years)	58.3 ± 4.8	59.3 ± 7.9	*0.406
Female sex (%)	38 (61.3%)	40 (60.6%)	^†^0.937
Hypertension (%)	13 (21.0%)	21 (31.8%)	^†^0.165
Right eye (%)	36 (58.1%)	34 (51.5%)	^†^0.457
UDVA (LogMAR)	0.24 ± 0.18	0.24 ± 0.20	*0.854
CDVA (LogMAR)	0.23 ± 0.21	0.24 ± 0.18	*0.701
UNVA (LogMAR)	0.35 ± 0.18	0.30 ± 0.18	*0.136
SphEq (D)	−1.32 ± 3.15	−1.53 ± 2.90	*0.695
Axial length (mm)	24.50 ± 1.46	24.26 ± 1.32	*0.330
WTW (mm)	11.97 ± 0.41	11.83 ± 0.45	*0.068
Corneal power (D)	43.56 ± 1.49	44.00 ± 1.57	*0.106
CCT (μm)	523.8 ± 27.6	515.6 ± 30.9	*0.115
IOL power (D)	18.6 ± 4.2	18.7 ± 3.8	*0.856

Continuous variables are reported as mean ± standard deviation; CCT = central corneal thickness; CT = conventional treatment; CDVA = corrected distance visual acuity; IOL = intraocular lens; LogMAR = logarithm of the minimum angle of resolution; PRP = platelet-rich plasma; SphEq = spherical equivalent; UDVA = uncorrected distance visual acuity; UNVA = uncorrected near visual acuity, WTW = white-to-white corneal diameter.

*P value by independent t-test.

^†^ *P* value by chi-square test.

### Optical quality parameters

**Fig 1:**
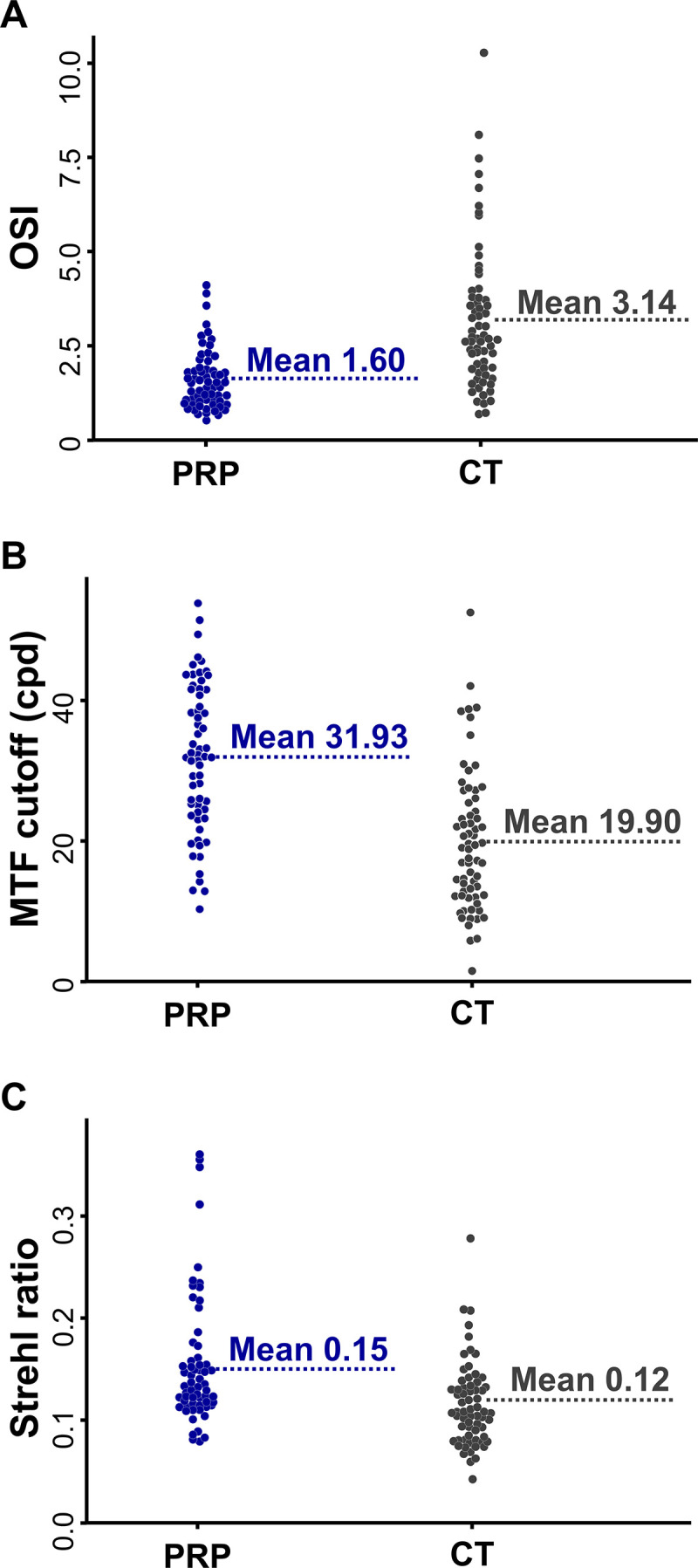
Distribution of the objective scatter index (OSI) (A), modulation transfer function (MTF) cutoff (B), and Strehl ratio (SR) (C) in eyes implanted with the CNWT intraocular lens, stratified by the postoperative use of autologous platelet-rich plasma (PRP) eye drops. The PRP group exhibited lower OSI and higher MTF cutoff and SR values, indicating superior optical performance compared to the conventional treatment (CT) group. cpd = cycles per degree.

The distribution of the OSI, MTF cutoff, and SR values measured at 3 weeks postoperatively are shown in Fig 1. The mean OSI was significantly lower in the PRP group than in the CT group (1.60 ± 0.80 vs. 3.14 ± 1.91, *P*< 0.001; Fig 1A). The mean MTF cutoff was significantly higher in the PRP group compared to the CT group (31.93 ± 10.52 vs. 19.90 ± 9.93 cycles per degree, *P* < 0.001; Fig 1B). Similarly, the mean SR was significantly higher in the PRP group than in the CT group (0.15 ± 0.06 vs. 0.12 ± 0.04, *P* < 0.001; Fig 1C).

### Non-invasive keratograph break-up time

**Fig 2:**
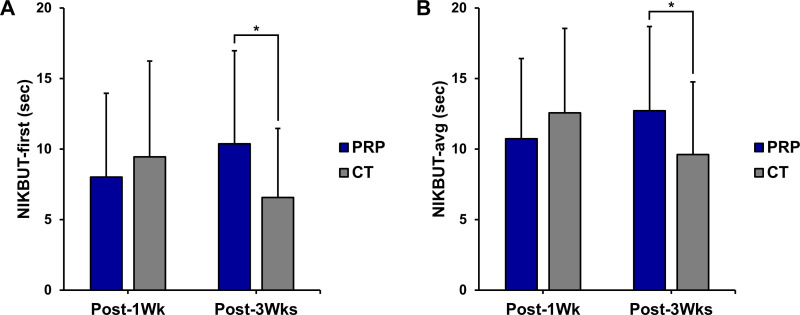
Noninvasive keratograph break-up time (NIKBUT) at 1 week and 3 weeks postoperatively. CT = conventional treatment group; NIKBUT-avg = average NIKBUT of all tear break-ups; NIKBUT-first = NIKBUT of the initial tear break-up; Post-1Wk = 1 week after the surgery; Post-3Wks = 3 weeks after the surgery; PRP = platelet-rich plasma group; sec = seconds. asterisk [*] denotes *P* < 0.05.

At the 1-week postoperative evaluation, the NIKBUT-first was 8.02 ± 5.94 seconds in the PRP group and 9.45 ± 6.80 seconds in the CT group, which was not a significant difference (*P* = 0.210). At 3 weeks postoperatively, the NIKBUT-first increased to 10.37 ± 6.60 seconds in the PRP group and decreased to 6.57 ± 4.90 seconds in the CT group, with a significant difference between the groups (*P* < 0.001; Fig 2A). Similarly, the NIKBUT-avg was 10.74 ± 5.69 seconds in the PRP group and 12.56 ± 5.99 seconds in the CT group at 1 week postoperatively (*P*= 0.080). By 3 weeks, the NIKBUT-avg increased to 12.72 ± 5.96 seconds in the PRP group, whereas it decreased to 9.62 ± 5.14 seconds in the CT group (*P* = 0.002, Fig 2B).

### Visual outcomes and intraocular lens position

The PRP group demonstrated superior visual acuity compared to the CT group. The mean logMAR UDVA was 0.05 ± 0.06 in the PRP group and 0.10 ± 0.10 in the CT group (*P* < 0.001). The mean logMAR CDVA was 0.01 ± 0.03 in the PRP group and 0.05 ± 0.07 in the CT group (*P* < 0.001). For UNVA, the mean values were 0.13 ± 0.10 in the PRP group and 0.18 ± 0.13 in the CT group (*P* = 0.007). No significant differences were observed between the two groups in terms of SphEq, corneal HOAs, SA, or the degree of IOL decentration and tilt (Table 2).

**Table 2:** Postoperative visual outcomes and intraocular lens position.

	PRP group (n = 62)	CT group (n = 66)	*P value
UDVA (LogMAR)	0.05 ± 0.06	0.10 ± 0.10	**< 0.001**
CDVA (LogMAR)	0.01 ± 0.03	0.05 ± 0.07	**< 0.001**
UNVA (LogMAR)	0.13 ± 0.10	0.18 ± 0.13	**0.007**
SphEq (D)	−0.21 ± 0.28	−0.26 ± 0.22	0.253
Higher-order aberration (μm)	0.25 ± 0.09	0.24 ± 0.06	0.296
Spherical aberration (μm)	0.25 ± 0.16	0.25 ± 0.13	0.894
IOL decentration (mm)	0.27 ± 0.16	0.28 ± 0.20	0.756
IOL tilt (°)	3.99 ± 1.46	4.01 ± 1.47	0.957

Continuous variables are reported as mean ± standard deviation; CDVA = corrected distance visual acuity; CT = conventional treatment; IOL = intraocular lens; LogMAR = logarithm of the minimum angle of resolution; PRP = platelet-rich plasma; SphEq = spherical equivalent; UDVA = uncorrected distance visual acuity; UNVA = uncorrected near visual acuity.

*P value by independent *t*-test.

### Factors associated with optical quality parameters

In the univariate analysis for OSI, PRP eye drop use and postoperative SphEq were identified as significant factors (*P* < 0.05) and were included in the multivariate analysis. In the multivariate regression analysis, only PRP eye drop use remained significantly associated with lower OSI values (B = –1.488 ± 0.261, *P* < 0.001; Table 3). Regarding the MTF cutoff and SR, PRP eye drop use was the only significant factor associated with higher MTF cutoff (B = 12.038 ± 1.808) and SR (B = 0.039 ± 0.009) values (*P* < 0.001), thus negating the need for further multivariate analysis (Table 3).

**Table 3:** Univariate and multivariate linear regression analysis for optical quality parameters.

	Objective scatter index				MTF cutoff		Strehl ratio	
	Univariate		Multivariate		Univariate		Univariate	
	*B (Std.err)	*P* value	*B (Std.err)	*P* value	*B (Std.err)	*P* value	*B (Std.err)	*P* value
PRP eye drop use	**−1.538 (0.262)**	**< 0.001**	**−1.488 (0.261)**	**< 0.001**	**12.038 (1.808)**	**< 0.001**	**0.039 (0.009)**	**< 0.001**
^†^NIKBUT-first (sec)	−0.039 (0.024)	0.107			0.315 (0.171)	0.069	0.000 (0.001)	0.917
^†^NIKBUT-avg (sec)	−0.044 (0.025)	0.089			0.215 (0.182)	0.241	0.000 (0.001)	0.928
Age	0.018 (0.023)	0.439			−0.185 (0.160)	0.250	−0.001 (0.001)	0.472
Female	0.162 (0.302)	0.592			1.491 (2.149)	0.489	0.013 (0.010)	0.192
Hypertension	−0.294 (0.333)	0.380			2.043 (2.371)	0.391	0.002 (0.011)	0.841
Axial length	0.042 (0.107)	0.695			−0.175 (0.758)	0.818	0.000 (0.004)	0.984
Preoperative SphEq (D)	−0.029 (0.049)	0.553			0.085 (0.350)	0.809	−0.001 (0.002)	0.637
WTW (mm)	−0.279 (0.339)	0.413			2.933 (2.405)	0.225	0.006 (0.012)	0.618
Corneal power (D)	−0.022 (0.096)	0.816			−0.096 (0.684)	0.889	0.001 (0.003)	0.808
CCT (μm)	−0.009 (0.005)	0.073			0.032 (0.036)	0.370	0.000 (0.000)	0.664
IOL power (D)	−0.008 (0.037)	0.825			0.071 (0.263)	0.788	0.000 (0.001)	0.830
Postoperative SphEq (D)	**−1.268 (0.575)**	**0.029**	−0.966 (0.517)	0.064	7.248 (4.117)	0.081	0.024 (0.020)	0.228
HOA (μm)	−1.738 (1.973)	0.380			7.242 (14.064)	0.607	−0.020 (0.068)	0.766
SA (μm)	−1.376 (1.012)	0.176			12.089 (7.168)	0.094	0.030 (0.035)	0.387
IOL decentration (mm)	−0.537 (0.799)	0.503			3.813 (5.684)	0.504	−0.023 (0.027)	0.398
IOL tilt (°)	−0.114 (0.101)	0.261			0.106 (0.724)	0.884	0.000 (0.003)	0.893

CCT = central corneal thickness; HOA = higher-order aberration; IOL = intraocular lens; MTF cutoff = modulation transfer function cutoff frequency; NIKBUT = non-invasive keratography break-up time; NIKBUT-avg = average NIKBUT of all tear break-ups; NIKBUT-first = NIKBUT of the initial tear break-up; PRP = platelet-rich plasma; SA = spherical aberration; SphEq = spherical equivalent; Std.err = standard error; WTW = white-to-white corneal diameter.

*Regression results are reported as the unstandardized regression coefficient B (standard error of B).

^†^ Values at postoperative 3 weeks.

Variance inflation factors for the multivariate model of objective scatter index were below 10, indicating no meaningful multicollinearity.

### Associations between optical quality parameters and postoperative visual acuity

Given the superior optical quality and visual acuity outcomes observed in the PRP group, further analyses were conducted to assess the relationship between optical quality parameters and postoperative visual acuity. Multivariate analysis revealed that improved optical quality was associated with better visual acuity outcomes. Specifically, lower OSI values, indicating better optical quality, were associated with better UDVA (B = 0.012 ± 0.004, *P* = 0.005), while higher MTF cutoff (B = −0.002 ± 0.001, *P* = 0.011) and SR (B = −0.251 ± 0.125, *P* = 0.047) values, also indicating better optical quality, were associated with better UDVA. Regarding CDVA, only OSI demonstrated a significant association (B = 0.007 ± 0.003, *P* = 0.028). For UNVA, both OSI (B = 0.019 ± 0.006, *P* = 0.002) and MTF cutoff (B = −0.002 ± 0.001, *P* = 0.010) showed significant correlations (Table 4).

**Table 4:** Univariate and multivariate linear regression analysis for postoperative visual acuity.

	Uncorrected distance visual acuity				Corrected distance visual acuity				Uncorrected near visual acuity			
	Univariate		*Multivariate		Univariate		^†^Multivariate		Univariate		^‡^Multivariate	
	^§^B (Std.err)	*P*	B (Std.err)	*P*	B (Std.err)	*P*	B (Std.err)	*P*	B (Std.err)	*P*	B (Std.err)	*P*
OSI	**0.014 (0.004)**	**0.002**	**0.012 (0.004)**	**0.005**	**0.008 (0.003)**	**0.007**	**0.007 (0.003)**	**0.028**	**0.021 (0.006)**	**0.001**	**0.019 (0.006)**	**0.002**
MTF cutoff	**−0.002 (0.001)**	**0.003**	**−0.002 (0.001)**	**0.011**	**−0.001 (0.000)**	**0.028**	−0.001 (0.000)	0.099	**−0.002 (0.001)**	**0.010**	**−0.002 (0.001)**	**0.010**
SR	**−0.267 (0.130)**	**0.042**	**−0.251 (0.125)**	**0.047**	−0.111 (0.093)	0.237	–	–	−0.159 (0.187)	0.396	–	–

MTF cutoff = modulation transfer function cutoff frequency; OSI = objective scatter index; SR = Strehl ratio; Std.err = standard error.

*Multivariate analysis adjusted for NIKBUT-first at postoperative 3 weeks and white-to-white corneal diameter.

^†^ Multivariate analysis adjusted for NIKBUT-first at postoperative 3 weeks, NIKBUT-avg at postoperative 3 weeks, and white-to-white corneal diameter.

^‡^ Multivariate analysis adjusted for sex and white-to-white corneal diameter.

^§^ Regression results are reported as the unstandardized regression coefficient B (standard error of B).

All variance inflation factors were below 10 in multivariate analyses, indicating no significant collinearity among included variables.

## Discussion

To the best of our knowledge, this is the first study to investigate the effect of autologous PRP eye drops on optical quality and visual outcomes after implantation of a trifocal diffractive IOL.

This study evaluated outcomes during the early postoperative period, 3 weeks after surgery. A functional magnetic resonance imaging study demonstrated that patients implanted with diffractive IOLs exhibited increased cortical activity at 3 weeks postoperatively [29], indicating the beginning of the neuroadaptation process to multifocal IOLs. Considering the critical role of the ocular surface and tear film in maintaining visual quality [30,31], ocular surface irregularities at this initial stage may negatively affect the neuroadaptation and subsequently impact long-term patient satisfaction [19,32,33]. Therefore, efforts to stabilize the ocular surface and optimize optical quality during the early postoperative period are particularly relevant following trifocal diffractive IOL implantation, especially in a preoperatively healthy population with elevated expectations. Future studies with longer follow-up periods are warranted to evaluate the sustained effects of PRP eye drops on optical quality and visual outcomes beyond the early postoperative phase.

To minimize the likelihood that postoperative differences in optical quality were confounded by pre-existing ocular surface abnormalities, the functional baseline was defined as the 1-week postoperative visit—the time point immediately before PRP initiation in the PRP group. All patients underwent comprehensive preoperative screening, and individuals with ocular surface disease or systemic conditions known to affect tear film stability (e.g., Sjögren syndrome, rheumatoid arthritis, thyroid dysfunction, or diabetes) were excluded [34]. Importantly, at this 1-week baseline visit, NIKBUT values did not significantly differ between the PRP and CT groups, indicating comparable tear film stability at the functional baseline. Although corneal staining was not assessed—representing a limitation of the study—the similarity in NIKBUT supports that both groups started from a comparable level of ocular surface integrity and dryness before the initiation of PRP therapy [26,27].

In this study, optical quality was assessed using OQAS parameters—including the OSI, MTF cutoff, and SR—based on earlier work demonstrating the utility of these measures for objectively evaluating optical quality in pseudophakic eyes [35–37]. Although direct comparisons are challenging, the optical quality parameters observed in the PRP group were comparable to those reported for monofocal IOLs in previous studies and even to those of individuals without lens opacity [22]. To better characterize the independent association between PRP eye drop use and optical quality, we included covariates such as IOL decentration [38], tilt [39] and tear break-up time [31,40] into multivariate analyses, as these factors are known to influence optical quality after multifocal IOL implantation. This approach enabled a more accurate characterization of the relationship between PRP eye drop use and optical quality, an area that has not been comprehensively addressed in previous studies [35–37].

Platelets are key reservoirs of growth factors, such as platelet-derived growth factor and epidermal growth factor, which are predominantly stored within their α-granules [9,10]. These growth factors promote tissue regeneration and healing, contributing to the broad therapeutic potential of PRP products. In ophthalmology, PRP has demonstrated promising effects in treating ocular surface diseases refractory to conventional therapies. The ocular surface restoration effects of PRP observed in previous studies are reflected in our findings as well.

While the platelet concentration of the PRP prepared in this study was not directly analyzed, the manufacturer’s technical brochure indicates that the 3E-PRP system yields a final platelet concentration approximately 9.3–12 times higher than the baseline whole-blood level [20]. PRP contains a variety of bioactive components beyond platelets, including multiple growth factors that play critical roles in tissue repair and ocular surface healing [10–12]. Future studies incorporating detailed compositional or proteomic analyses—including quantification of platelet concentration and characterization of growth factors and other bioactive molecule profiles—will be valuable to elucidate the components strongly associated with clinical efficacy and to establish standardized preparation and dosing protocols for ophthalmic PRP applications.

The PRP group demonstrated a significant improvement in NIKBUT at 3 weeks postoperatively, whereas earlier reports—consistent with findings in the CT group—have demonstrated a decrease in tear break-up time after cataract surgery [41,42]. Despite this improvement in NIKBUT, no direct association with optical quality parameters was found in this study. This may be because NIKBUT does not fully capture spatial variability in tear film stability. The impact on optical quality in eyes with trifocal diffractive IOLs may vary depending on whether tear film perturbations occur centrally or peripherally, a distinction not accounted for by NIKBUT. Moreover, factors such as corneal morphology and transparency also influence optical quality [43,44]. Therefore, further studies incorporating more spatially detailed tear film assessments and corneal evaluations are warranted to elucidate the mechanisms underlying the improvements observed in the PRP group.

Multivariate analyses revealed significant correlations between the OQAS parameters and visual acuity outcomes. This is consistent with the observation that the PRP group exhibited superior optical quality and visual acuity compared with the CT group. Previous studies have also demonstrated significant associations between OQAS parameters and visual acuity, supporting the findings of the present investigation [45–47].

All patients in this study underwent cataract surgery with a standardized femtosecond laser capsulotomy size and a uniform quadrant nuclear fragmentation pattern. This standardization is noteworthy, as the size and shape of the capsulotomy can affect IOL positioning [48], potentially influencing optical quality outcomes. Such procedural uniformity enhances the internal validity of our study.

Although the present study consecutively enrolled all eligible patients who underwent FLACS with CNWT IOL implantation during the study period, the relatively strict exclusion criteria limit the external validity of the results. These criteria reflect the narrow indication for multifocal IOL implantation, which is generally restricted to eyes without ocular or systemic comorbidities that may adversely affect postoperative visual performance [1,8]. Furthermore, exclusions relevant to the safe or appropriate application of autologous PRP—such as the current use of antiplatelet agents and infectious status including hepatitis B carrier state—were applied equally to both groups to maintain comparability and reduce potential confounding [49,50]. As a result, the study population represents a highly selected subset of cataract surgery patients; however, this approach was necessary to maintain cohort homogeneity and minimize confounding factors when evaluating early postoperative ocular surface and optical quality outcomes.

It is crucial to acknowledge the following limitations when interpreting our findings. First, the retrospective, non-randomized design introduces inherent constraints. Although baseline characteristics were similar between the PRP and CT groups and multivariate analyses were performed to control for confounders, unmeasured variables—such as socioeconomic status, lifestyle factors, and adherence to postoperative regimens—may have influenced treatment allocation and outcomes. Future randomized controlled trials are needed to validate these findings. Second, corneal staining was not evaluated. While NIKBUT provides a non-invasive measure of tear film stability and avoids dye-induced reflex tearing, corneal staining could offer additional insight into epithelial integrity and its impact on optical quality. Third, PRP eye drops were used alongside antibiotics, steroid eye drops, and HA artificial tears; the improvements observed should therefore be considered additive rather than solely attributable to PRP. Fourth, the follow-up duration was relatively short, which may not fully capture the long-term stability of ocular surface recovery or the persistence of optical quality improvements. Longer-term studies are needed to determine whether the early benefits of PRP are sustained over time. Fifth, patient-reported outcomes were not collected. Instruments such as the Ocular Surface Disease Index [51] or visual quality questionnaires [52] would provide complementary subjective information and help contextualize the clinical relevance of the optical improvements observed. Future studies that integrate both subjective and objective measures will enhance the generalizability and translational value of these findings.

In conclusion, autologous PRP eye drops improve optical quality and visual outcomes following trifocal diffractive IOL implantation. Further studies incorporating detailed corneal surface assessments and longer follow-up periods are warranted to provide deeper insights into the effects of PRP on optical and visual performance in eyes with trifocal diffractive IOLs.

## References

[1] Rampat R, Gatinel D. Multifocal and extended depth-of-focus intraocular lenses in 2020. Ophthalmology. 2021;128(11):e164–85. doi: 10.1016/j.ophtha.2020.09.026 32980397

[2] Kohnen T, Herzog M, Hemkeppler E, Schönbrunn S, De Lorenzo N, Petermann K, et al. Visual Performance of a quadrifocal (trifocal) intraocular lens following removal of the crystalline lens. Am J Ophthalmol. 2017;184:52–62. doi: 10.1016/j.ajo.2017.09.016 28923587

[3] Bilbao-Calabuig R, Llovet-Rausell A, Ortega-Usobiaga J, Martínez-Del-Pozo M, Mayordomo-Cerdá F, Segura-Albentosa C, et al. Visual outcomes following bilateral lmplantation of two diffractive trifocal intraocular lenses in 10 084 eyes. Am J Ophthalmol. 2017;179:55–66. doi: 10.1016/j.ajo.2017.04.013 28456547

[4] Brenner LF, Nistad K, Schonbeck U. Rethinking presbyopia: results of bilateral refractive lens exchange with trifocal intraocular lenses in 17 603 patients. Br J Ophthalmol. 2023;107(7):912–9. doi: 10.1136/bjophthalmol-2021-319732 35110276

[5] Na K-S, Kim S-J, Nam G, Ha M, Whang W-J, Kim EC, et al. A novel intraocular lens simulator that allows patients to experience the world through multifocal intraocular lenses before surgeries. Transl Vis Sci Technol. 2022;11(3):14. doi: 10.1167/tvst.11.3.14 35275206 PMC8934550

[6] Kim EC, Na K-S, Kim HS, Hwang HS. How does the world appear to patients with multifocal intraocular lenses?: A mobile model eye experiment. BMC Ophthalmol. 2020;20(1):180. doi: 10.1186/s12886-020-01446-5 32375711 PMC7201983

[7] Kim EC, Cho SY, Kang JE, Nam G, Yoon YC, Whang W-J, et al. Comparative analysis of optical quality of monofocal, enhanced monofocal, multifocal, and extended depth of focus intraocular lenses: A mobile model eye study. Transl Vis Sci Technol. 2023;12(7):5. doi: 10.1167/tvst.12.7.5 37405796 PMC10327964

[8] Alio JL, Plaza-Puche AB, Férnandez-Buenaga R, Pikkel J, Maldonado M. Multifocal intraocular lenses: An overview. Surv Ophthalmol. 2017;62(5):611–34. doi: 10.1016/j.survophthal.2017.03.005 28366683

[9] Anitua E, Muruzabal F, Tayebba A, Riestra A, Perez VL, Merayo-Lloves J, et al. Autologous serum and plasma rich in growth factors in ophthalmology: Preclinical and clinical studies. Acta Ophthalmol. 2015;93(8):e605-14. doi: 10.1111/aos.12710 25832910

[10] Nurden AT. Platelets, inflammation and tissue regeneration. Thromb Haemost. 2011;105 Suppl 1:S13-33. doi: 10.1160/THS10-11-0720 21479340

[11] Kim KM, Shin Y-T, Kim HK. Effect of autologous platelet-rich plasma on persistent corneal epithelial defect after infectious keratitis. Jpn J Ophthalmol. 2012;56(6):544–50. doi: 10.1007/s10384-012-0175-y 22972393

[12] Alio JL, Rodriguez AE, WróbelDudzińska D. Eye platelet-rich plasma in the treatment of ocular surface disorders. Curr Opin Ophthalmol. 2015;26(4):325–32. doi: 10.1097/ICU.0000000000000169 26058033

[13] Metheetrairut C, Ngowyutagon P, Tunganuntarat A, Khowawisetsut L, Kittisares K, Prabhasawat P. Comparison of epitheliotrophic factors in platelet-rich plasma versus autologous serum and their treatment efficacy in dry eye disease. Sci Rep. 2022;12(1):8906. doi: 10.1038/s41598-022-12879-x 35618742 PMC9135723

[14] Kang M-J, Lee JH, Hwang J, Chung S-H. Efficacy and safety of platelet-rich plasma and autologous-serum eye drops for dry eye in primary Sjögren’s syndrome: A randomized trial. Sci Rep. 2023;13(1):19279. doi: 10.1038/s41598-023-46671-2 37935760 PMC10630514

[15] Wróbel-Dudzińska D, Przekora A, Kazimierczak P, Ćwiklińska-Haszcz A, Kosior-Jarecka E, Żarnowski T. The comparison between the composition of 100% autologous serum and 100% platelet-rich plasma eye drops and their impact on the treatment effectiveness of dry eye disease in primary sjogren syndrome. J Clin Med. 2023;12(9):3126. doi: 10.3390/jcm12093126 37176566 PMC10179661

[16] Alio JL, Abad M, Artola A, Rodriguez-Prats JL, Pastor S, Ruiz-Colecha J. Use of autologous platelet-rich plasma in the treatment of dormant corneal ulcers. Ophthalmology. 2007;114(7):1286-1293.e1. doi: 10.1016/j.ophtha.2006.10.044 17324465

[17] Alio JL, Rodriguez AE, Abdelghany AA, Oliveira RF. Autologous platelet-rich plasma eye drops for the treatment of post-LASIK chronic ocular surface syndrome. J Ophthalmol. 2017;2017:2457620. doi: 10.1155/2017/2457620 29379652 PMC5742891

[18] Gibbons A, Ali TK, Waren DP, Donaldson KE. Causes and correction of dissatisfaction after implantation of presbyopia-correcting intraocular lenses. Clin Ophthalmol. 2016;10:1965–70. doi: 10.2147/OPTH.S114890 27784985 PMC5066995

[19] Woodward MA, Randleman JB, Stulting RD. Dissatisfaction after multifocal intraocular lens implantation. J Cataract Refract Surg. 2009;35(6):992–7. doi: 10.1016/j.jcrs.2009.01.031 19465282 PMC5125020

[20] Pervice Co., Ltd. 3E-PRP System Technical Brochure. Includes manufacturer-provided preparation protocol and platelet concentration data (based on Green Cross Medical Foundation platelet count testing). https://oyasama.es/wp-content/uploads/2022/02/3E-PRP-brochure-NEW.pdf

[21] Güell JL, Pujol J, Arjona M, Diaz-Douton F, Artal P. Optical quality analysis system; Instrument for objective clinical evaluation of ocular optical quality. J Cataract Refract Surg. 2004;30(7):1598–9. doi: 10.1016/j.jcrs.2004.04.031 15210251

[22] Saad A, Saab M, Gatinel D. Repeatability of measurements with a double-pass system. J Cataract Refract Surg. 2010;36(1):28–33. doi: 10.1016/j.jcrs.2009.07.033 20117702

[23] Cabot F, Saad A, McAlinden C, Haddad NM, Grise-Dulac A, Gatinel D. Objective assessment of crystalline lens opacity level by measuring ocular light scattering with a double-pass system. Am J Ophthalmol. 2013;155(4):629–35, 635.e1-2. doi: 10.1016/j.ajo.2012.11.005 23317652

[24] Díaz-Doutón F, Benito A, Pujol J, Arjona M, Güell JL, Artal P. Comparison of the retinal image quality with a Hartmann-Shack wavefront sensor and a double-pass instrument. Invest Ophthalmol Vis Sci. 2006;47(4):1710–6. doi: 10.1167/iovs.05-1049 16565413

[25] Artal P, Benito A, Pérez GM, Alcón E, De Casas A, Pujol J, et al. An objective scatter index based on double-pass retinal images of a point source to classify cataracts. PLoS One. 2011;6(2):e16823. doi: 10.1371/journal.pone.0016823 21326868 PMC3033912

[26] Hong J, Sun X, Wei A, Cui X, Li Y, Qian T, et al. Assessment of tear film stability in dry eye with a newly developed keratograph. Cornea. 2013;32(5):716–21. doi: 10.1097/ICO.0b013e3182714425 23132457

[27] Tian L, Qu J-H, Zhang X-Y, Sun X-G. Repeatability and reproducibility of noninvasive keratograph 5M measurements in patients with dry eye disease. J Ophthalmol. 2016;2016:8013621. doi: 10.1155/2016/8013621 27190639 PMC4844888

[28] Dormann CF, Elith J, Bacher S, Buchmann C, Carl G, Carré G, et al. Collinearity: A review of methods to deal with it and a simulation study evaluating their performance. Ecography. 2012;36(1):27–46. doi: 10.1111/j.1600-0587.2012.07348.x

[29] Rosa AM, Miranda ÂC, Patrício MM, McAlinden C, Silva FL, Castelo-Branco M, et al. Functional magnetic resonance imaging to assess neuroadaptation to multifocal intraocular lenses. J Cataract Refract Surg. 2017;43(10):1287–96. doi: 10.1016/j.jcrs.2017.07.031 29120714

[30] Koh S. Mechanisms of visual disturbance in dry eye. Cornea. 2016;35 Suppl 1:S83–8. doi: 10.1097/ICO.0000000000000998 27583799

[31] Herbaut A, Liang H, Rabut G, Trinh L, Kessal K, Baudouin C, et al. Impact of dry eye disease on vision quality: An optical quality analysis system study. Transl Vis Sci Technol. 2018;7(4):5. doi: 10.1167/tvst.7.4.5 30009091 PMC6042522

[32] Alió JL, Pikkel J. Multifocal intraocular lenses: Neuroadaptation. Alió JL, Pikkel J. Multifocal Intraocular Lenses. Springer. 2019. 53–60.

[33] de Vries NE, Webers CAB, Touwslager WRH, Bauer NJC, de Brabander J, Berendschot TT, et al. Dissatisfaction after implantation of multifocal intraocular lenses. J Cataract Refract Surg. 2011;37(5):859–65. doi: 10.1016/j.jcrs.2010.11.032 21397457

[34] Qian L, Wei W. Identified risk factors for dry eye syndrome: A systematic review and meta-analysis. PLoS One. 2022;17(8):e0271267. doi: 10.1371/journal.pone.0271267 35984830 PMC9390932

[35] Jiménez R, Valero A, Fernández J, Anera RG, Jiménez JR. Optical quality and visual performance after cataract surgery with biaxial microincision intraocular lens implantation. J Cataract Refract Surg. 2016;42(7):1022–8. doi: 10.1016/j.jcrs.2016.03.039 27492101

[36] Liao X, Li J-Y, Tan Q-Q, Tian J, Lin J, Lan C-J. Comparison of visual quality after implantation of A1-UV and SN60WF aspheric intraocular lens. Int J Ophthalmol. 2020;13(11):1727–32. doi: 10.18240/ijo.2020.11.07 33215002 PMC7590885

[37] Lee H, Lee K, Ahn JM, Kim EK, Sgrignoli B, Kim T-I. Double-pass system assessing the optical quality of pseudophakic eyes. Optom Vis Sci. 2014;91(4):437–43. doi: 10.1097/OPX.0000000000000190 24492759

[38] Ortiz C, Esteve-Taboada JJ, Belda-Salmerón L, Monsálvez-Romín D, Domínguez-Vicent A. Effect of decentration on the optical quality of two intraocular lenses. Optom Vis Sci. 2016;93(12):1552–9. doi: 10.1097/OPX.0000000000001004 27776082

[39] Oshika T, Sugita G, Miyata K, Tokunaga T, Samejima T, Okamoto C, et al. Influence of tilt and decentration of scleral-sutured intraocular lens on ocular higher-order wavefront aberration. Br J Ophthalmol. 2007;91(2):185–8. doi: 10.1136/bjo.2006.099945 16914469 PMC1857623

[40] Gao Y, Liu R, Liu Y, Ma B, Yang T, Hu C, et al. Optical quality in patients with dry eye before and after treatment. Clin Exp Optom. 2021;104(1):101–6. doi: 10.1111/cxo.13111 32618024

[41] Kasetsuwan N, Satitpitakul V, Changul T, Jariyakosol S. Incidence and pattern of dry eye after cataract surgery. PLoS One. 2013;8(11):e78657. doi: 10.1371/journal.pone.0078657 24265705 PMC3827040

[42] Shimabukuro M, Maeda N, Koh S, Abe K, Kobayashi R, Nishida K. Effects of cataract surgery on symptoms and findings of dry eye in subjects with and without preexisting dry eye. Jpn J Ophthalmol. 2020;64(4):429–36. doi: 10.1007/s10384-020-00744-1 32495157

[43] McCally RL, Farrell RA. Light Scattering from Cornea and Corneal Transparency. Noninvasive Diagnostic Techniques in Ophthalmology. Springer New York. 1990. 189–210. doi: 10.1007/978-1-4613-8896-8_12

[44] Meek KM. Corneal collagen-its role in maintaining corneal shape and transparency. Biophys Rev. 2009;1(2):83–93. doi: 10.1007/s12551-009-0011-x 28509987 PMC5425665

[45] Martinez-Roda JA, Vilaseca M, Ondategui JC, Almudi L, Asaad M, Mateos-Pena L, et al. Double-pass technique and compensation-comparison method in eyes with cataract. J Cataract Refract Surg. 2016; 42:1461–9. doi: 10.1016/j.jcrs.2016.08.015. 27839601

[46] Martínez-Roda JA, Vilaseca M, Ondategui JC, Aguirre M, Pujol J. Effects of aging on optical quality and visual function. Clin Exp Optom. 2016;99(6):518–25. doi: 10.1111/cxo.12369 27452417

[47] Li Y, Jin L, Wu M, Huang Y. Evaluation value of subjective visual quality examination on surgical indications of the early cataracts based on objective scatter index values. Front Med (Lausanne). 2022;9:1075693. doi: 10.3389/fmed.2022.1075693 36582278 PMC9792837

[48] Rossi T, Ceccacci A, Testa G, Ruggiero A, Bonora N, D’Agostino I, et al. Influence of anterior capsulorhexis shape, centration, size, and location on intraocular lens position: finite element model. J Cataract Refract Surg. 2022;48(2):222–9. doi: 10.1097/j.jcrs.0000000000000711 34117178 PMC8845527

[49] Wu PI-K, Diaz R, Borg-Stein J. Platelet-rich plasma. Phys Med Rehabil Clin N Am. 2016;27(4):825–53. doi: 10.1016/j.pmr.2016.06.002 27788903

[50] Eymard F, Louati K, Noel É, Abouqal R, Adam P, Allali F, et al. Indications and contraindications to platelet-rich plasma injections in musculoskeletal diseases in case of infectious, oncological and haematological comorbidities: A 2025 formal consensus from the GRIIP (International Research Group on Platelet Injections). Knee Surg Sports Traumatol Arthrosc. 2025;33(6):2293–306. doi: 10.1002/ksa.12682 40260684 PMC12104799

[51] Pult H, Wolffsohn JS. The development and evaluation of the new Ocular Surface Disease Index-6. Ocul Surf. 2019;17(4):817–21. doi: 10.1016/j.jtos.2019.08.008 31442595

[52] McAlinden C, Pesudovs K, Moore JE. The development of an instrument to measure quality of vision: The Quality of Vision (QoV) questionnaire. Invest Ophthalmol Vis Sci. 2010;51(11):5537–45. doi: 10.1167/iovs.10-5341 20505205

